# A risk stratification model modified from the U.S. guideline could be applied in an Asian population with or without ASCVD: Validation study

**DOI:** 10.1016/j.bj.2023.100653

**Published:** 2023-08-12

**Authors:** Yu-Chung Hsiao, Thung-Lip Lee, Fang-Ju Lin, Chin-Feng Hsuan, Chih-Fan Yeh, Wei-Tien Chang, Hsien-Li Kao, Jiann-Shing Jeng, Yen-Wen Wu, I-Chang Hsieh, Ching-Chang Fang, Kuo-Yang Wang, Kuan-Cheng Chang, Tsung-Hsien Lin, Wayne Huey-Herng Sheu, Yi-Heng Li, Wei-Hsian Yin, Hung-I Yeh, Jaw-Wen Chen, Chau-Chung Wu, Taiwanese Secondary and Primary Prevention for Patients with AtheRosCLErotic Disease (T-SPARCLE & T-PPARCLE) Registries Investigators

**Affiliations:** a Department of Internal Medicine, National Taiwan University Hospital, Taipei, Taiwan; b Division of Cardiology, Department of Internal Medicine, E-Da Hospital, Kaohsiung, Taiwan; c School of Medicine, College of Medicine, I-Shou University, Kaohsiung, Taiwan; d Graduate Institute of Clinical Pharmacy & School of Pharmacy, College of Medicine, National Taiwan University, Taipei, Taiwan; e Department of Pharmacy, National Taiwan University Hospital, Taipei, Taiwan; f Division of Cardiology, Department of Internal Medicine, E-Da Dachang Hospital, Kaohsiung, Taiwan; g Division of Cardiology, Department of Internal Medicine and Cardiovascular Center, National Taiwan University Hospital, Taipei, Taiwan; h Department of Emergency Medicine, National Taiwan University Hospital, Taipei, Taiwan; i Department of Neurology, National Taiwan University Hospital, Taipei, Taiwan; j Division of Cardiology, Cardiovascular Medical Center, Far Eastern Memorial Hospital, New Taipei, Taiwan; k Division of Cardiology, Department of Internal Medicine, Chang Gung Memorial Hospital at Linkou, Taoyuan, Taiwan; l College of Medicine, Chang Gung University, Taoyuan, Taiwan; m Division of Cardiology, Tainan Municipal Hospital, Tainan, Taiwan; n Division of Cardiology, Taichung Veterans General Hospital, Taichung, Taiwan; o Division of Cardiology, Department of Internal Medicine, China Medical University Hospital, Taichung, Taiwan; p Division of Cardiology, Department of Internal Medicine, Kaohsiung Medical University Hospital, Kaohsiung, Taiwan; q Faculty of Medicine, College of Medicine, Kaohsiung Medical University, Kaohsiung, Taiwan; r Division of Endocrinology and Metabolism and Department of Internal Medicine, Taipei Veterans General Hospital, Taichung, Taiwan; s Division of Cardiology, Department of Internal Medicine, National Cheng Kung University Hospital, Tainan, Taiwan; t Division of Cardiology, Heart Center, Cheng-Hsin General Hospital, Taipei, Taiwan; u Mackay Memorial Hospital, Mackay Medical College, Taipei, Taiwan; v Department of Medical Research and Education, Taipei Veterans General Hospital, Taipei, Taiwan; w Graduate Institute of Medical Education & Bioethics, College of Medicine, National Taiwan University, Taipei, Taiwan

**Keywords:** ASCVD, Risk model, Cohort study

## Abstract

**Background:**

This study aimed to evaluate the performance of a modified U.S. (MUS) model for risk prediction of cardiovascular (CV) events in Asian patients and compare it to European and Japanese models.

**Methods:**

The MUS model, based on the US ACC/AHA 2018 lipid treatment guideline, was employed to stratify patients under primary or secondary prevention. Two multi-center prospective observational registry cohorts, T-SPARCLE and T-PPARCLE, were used to validate the scoring system, and the primary outcome was the time to first occurrence/recurrence of major adverse cardiac events (MACEs). The MUS model's performance was compared to other models from Europe and Japan.

**Results:**

A total of 10,733 patients with the mean age of 64.2 (SD: 11.9) and 36.5% female were followed up for a median of 5.4 years. The MUS model was validated, with an AUC score of 0.73 (95% CI 0.68–0.78). The European and Japanese models had AUC scores ranging from 0.6 to 0.7. The MUS model categorized patients into four distinct CV risk groups, with hazard ratios (HRs) as follows: very high- vs. high-risk group (HR = 1.91, 95% CI 1.53–2.39), high- vs. moderate-risk group (HR = 2.08, 95% CI 1.60–2.69), and moderate- vs. low-risk group (HR = 3.14, 95% CI 1.63–6.03). After adjusting for the MUS model, a history of atherosclerotic vascular disease (ASCVD) was not a significant predictor of adverse cardiovascular outcomes within each risk group.

**Conclusion:**

The MUS model is an effective tool for risk stratification in Asian patients with and without ASCVD, accurately predicting MACEs and performing comparably or better than other established risk models. Our findings suggest that patient management should focus on background risk factors instead of solely on primary or secondary prevention.

**At a glance commentary**

**Scientific background on the subject**

Cardiovascular disease ranks among the top global causes of death, characterized by distinct epidemiological patterns in East Asia. Despite many risk models originating from Western research, limited focus has been placed on their suitability for Asian populations. Our research aims to develop a universally applicable, straightforward cardiovascular risk prediction model for Asians, drawing inspiration from the health belief model and taking into account common human physiology and global lifestyle factors.

**What this study adds to the field**

The MUS model, derived from the 2018 US ACC/AHA guideline, emerged as a valuable risk assessment tool for Asian patients, whether they had ASCVD or not. It displayed strong predictive accuracy for cardiovascular events. Its simplicity and reliance on readily accessible parameters render it a practical choice for both Asian patients and healthcare providers. This study underscores the importance of tailoring patient management to individual risk factors, transcending the traditional primary and secondary prevention approach.

## Introduction

Cardiovascular (CV) disease is a leading cause of death worldwide [1]. The epidemiology of cardiovascular events in East Asia differs slightly from that of many western countries, with stroke being more prevalent than coronary artery disease (CAD), and higher rates of hypertension and lower cholesterol levels [2]. Despite the difference, the overall treatment approach remains largely the same. For instance, the Taiwanese guideline for managing dyslipidemia has many similarities to the guidelines used in the US and Europe [[3], [4], [5]]. Many studies have examined the effectiveness of western atherosclerotic vascular disease (ASCVD) risk assessment models in identifying at-risk Asian populations [[6], [7], [8], [9]], while less attention has been given to those who already have ASCVD.

The application of an intuitive risk calculation model holds considerable promise in bolstering patient health literacy, which can have a profound ripple effect on positively transforming their health behaviors [10,11]. Drawing upon the principles of the health belief model [12], this knowledge acquisition and behavior change could potentiate superior clinical outcomes, especially when the model is easily comprehensible and readily accessible. The simplicity of a model is often a determining factor in its usability and consequent effectiveness. With this understanding, our study was guided by the intent to deploy a straightforward yet robust model for risk prediction within our cohorts. We moved beyond the traditional geographical confines of these models, hypothesizing that well-established Western models may transcend cultural and regional differences. The potential for these models to be equally applicable for both primary and secondary prevention in the Asian demographic forms the cornerstone of our research. This hypothesis is not only rooted in the universality of human physiology but also underpinned by the increasing global convergence of lifestyle factors that contribute to cardiovascular risk.

Therefore, our study seeks to bridge this gap, potentially paving the way for more universally applicable healthcare strategies. We aimed to validate a very simple CV scoring system modified from the U.S. guideline in an Asian population. Other well-known CV scoring systems from Europe and Japan were also examined.

## Material and methods

### Data source

The study population included men and women aged >18 years who were enrolled in the two registries, the Taiwanese Secondary Prevention for patients with AtheRosCLErotic disease (T-SPARCLE) Registry and Taiwanese Primary Prevention for AtheRosCLErotic disease (T-PPARCLE) Registry. Both of the registries enrolled patients from 14 medical centers across Taiwan:

(a) The T-SPARCLE registry followed patients with ASCVD. Enrolled patients were those with significant coronary artery occlusion >50% in diameter, which was identified by a cardiac catheterization examination, having a history of myocardial infarction (MI) as evidenced by electrocardiography or hospitalization, or angina with ischemic changes or positive response to stress test. Patients with cerebral vascular disease, defined as cerebral infarction, intracerebral hemorrhage (excluding intracerebral hemorrhage associated with trauma or other diseases), and transient ischemic attack whose ultrasound confirms atheromatous change with >70% blockage in the carotid artery, were enrolled. Patients with peripheral atherosclerosis with symptoms of ischemia and confirmed by ankle-brachial index, Doppler ultrasound, or angiography were also enrolled.
(b) The T-PPARCLE registry followed patients with no evidence of ASCVD, but with at least one of the following risk factors: diabetes mellitus (DM), dyslipidemia, hypertension, smoking, elder age (men >45 years old, women >55 years old), family history of premature CAD (men <55 years old, women <65 years old), and obesity (waist circumference: men >90 cm, women >80 cm). Patients were defined as having dyslipidemia if one of the following criteria was met: under lipid-lowering therapy, total cholesterol (TC) >200 mg/dL; LDL-C >130 mg/dL; TG >200 mg/dL; men with HDL-C <40 mg/dL or women with HDL-C <50 mg/dL.

Patients with neuro-cognitive or psychiatric condition, end-stage renal disease on dialysis, serious heart disease with functional class III or IV heart failure, life expectancy shorter than 6 months, or treatment with immunosuppressive agents were excluded. Patients with recent acute stroke, acute MI, undergoing coronary revascularization within 3 months were also excluded. Patients who were enrolled in the study participated in annual follow-up appointments at the respective clinics. In circumstances warranting additional contact, our team would reach out via phone to ensure a comprehensive monitoring of their health status. Clinical outcomes, laboratory data and medication use were recorded at enrollment and each follow-up. The clinical outcomes included MACE, non-cardiovascular death, congestive heart failure, peripheral arterial disease. Smoking status, physical activity, and other concomitant secondary prevention interventions were also recorded.

In this study, patients were categorized into two groups based on the registries: those with ASCVD and those without ASCVD.

#### Modified U.S. (MUS) ACC/AHA model

According to the ACC/AHA 2018 guidelines for managing blood cholesterol in the United States, individuals with ≥2 major risk factors or 1 major risk factor plus ≥2 minor risk factors are classified as very high-risk patients and are recommended to undergo high-intensity lipid management [3]. Based on this recommendation, we developed the modified U.S. model as in[Table 1].

**Table 1:** The development of the modified U.S. model.

Original 2018 ACC/AHA guideline	Modified U.S. model
**Major risk factors**	**+2 points if any:**
Recent acute coronary syndrome (ACS) (within the past 12 months)	History of acute coronary syndrome (ACS)
History of myocardial infarction (other than recent ACS event listed above)	
History of cerebrovascular disease	History of cerebrovascular disease
Symptomatic peripheral arterial disease (history of claudication with ankle brachial index <0.85, or previous revascularization or amputation)	History of peripheral artery disease
**Minor risk factors**	**+1 point if any:**
Age ≥65 years old	Age ≥65 years old
Persistently elevated LDL-C (LDL-C ≥100 mg/dL [≥2.6 mmol/L]) despite maximally tolerated statin therapy and ezetimibe	Under statin and/or ezetimibe, LDL-C level still >100 mg/dL if having a history of ASCVD or LDL-C level still >130 mg/dL if not having a history of ASCVD
Hypertension	Hypertension
Diabetes mellitus	Diabetes mellitus
Chronic kidney disease CKD (eGFR 15–59 mL/min/1.73 m^2^)	Chronic kidney disease (CKD), stage 3 or greater
Congestive heart failure (HF)	Heart failure (HF)
History of prior coronary artery bypass surgery or percutaneous coronary intervention outside of the major ASCVD event(s)	History of CAD in those without a history of ACS
Current smoker	Current smoker
Heterozygous familial hypercholesterolemia	–

*Other validated CV scoring systems tested in the cohorts*:

1. SMART2 (Secondary Manifestations of ARTerial disease) [13,14]: The model was originally developed from a Netherlands ASCVD Cohort. In this study, it was used in both with or without ASCVD. However, due to the unavailability of certain data in our cohort, we made some modifications to the model. Specifically, we excluded abdominal aneurysm, C-reactive protein (CRP) levels, and years since ASCVD diagnosis as these variables were not available in our dataset. Instead, we incorporated the following parameters in our adapted model, while preserving the original coefficients: age, sex, coronary artery disease (CAD), peripheral arterial occlusive disease (PAOD), estimated glomerular filtration rate (eGFR), systolic blood pressure, diabetes mellitus (DM), non-high-density lipoprotein cholesterol (non-HDL-C), current smoking status, and antiplatelet use.
2. European Society of Cardiology SCORE2 (for age 40–69) and SCORE2-OP (for age ≥70) [15]: The model was initially developed for those without ASCVD and aged 40–89. There were high and low CVD risk panels provided by the SCORE2 model, and the low CVD risk panel was used in this study based on the epidemiology in Taiwan [16,17].
3. Japan model [18]: The model was developed from the Hisayama Study, a cohort without ASCVD. However, due to the unavailability of certain data in our cohort, we made some modifications for the model. Specifically, we excluded proteinuria and exercise. Instead, we incorporated the following parameters in our adapted model, while preserving the original coefficients: age, sex, systolic blood pressure, DM, HDL-C, LDL-C, current smoking.

### Outcomes

The primary outcome of this study was MACE, which included CV death, hospitalization for nonfatal MI or stroke, or cardiac arrest with resuscitation during the follow-up period. Time to the first occurrence/recurrence of MACE was studied. Participants who died from non-CV causes were included and censored at the date of the non-CV death event. Otherwise, participants were censored at the last follow up date.

### Statistical analysis

Categorical variables were presented in percentage, and continuous or discrete variables are presented as mean and standard deviation. The chi-square test was used to compare proportions; student's t-test was used to compare differences in continuous variables between groups. Statistical analyses were performed using the SAS 9.4 software (SAS Institute Inc., Cary, NC).

The models were used to predict the outcome within 1 year (MACE or without MACE). The performance of each model was also evaluated by the area under the receiver operating characteristic curve (AUC). We used SAS PROC LOGISTIC procedure to compute AUC and Brier score. The Brier score is a metric used to evaluate the accuracy of probabilistic predictions, providing a holistic measure of a model's performance. A lower Brier score signifies a model with better predictive accuracy. We also used SAS PROC LOGISTIC procedure with LACKFIT for Hosmer–Lemeshow goodness-of-fit Test. PROC LOGISTIC along with a data step and PROC SGPLOT was used for creation of calibration plot. Four scoring systems were calculated for each suitable individual. The imputed dataset was used to validate the performance of various models.

Patients were divided into four CV risk groups based on the results of the modified US (MUS) model. Further survival analysis during follow up time were performed with SAS COX PHREG. The probability of MACE occurrence in each CV risk group was presented using Kaplan Meier methods, and the differences among groups were compared using Cox regression model. The proportional hazards assumption was tested for each multivariable Cox regression model.

Cox regression model was used to estimate the hazard ratios (HRs) and 95% confidence intervals (CIs) for MACE outcome. *p*-value <0.05 was considered statistically significant. We used multiple imputation (PROC MI procedure in SAS) to handle missing values. The imputation step resulted in 20 complete data sets, each containing unique imputed values for the missing data. After imputation, we fitted Cox proportional hazards model for each dataset and then used PROC MIANALYZE procedure in SAS to combine results from each Cox model.

### Ethics statement

The study was approved by the Joint Institutional Review Board (JIRB), Taiwan, R.O.C. (number: 09-S-015) or hospital IRB for each participating hospital. Written informed consent was obtained from all the enrolled patients.

## Results

Patients were enrolled from December, 2009 to August, 2019. A total of 10,733 patients with the mean age of 64.2 (SD: 11.9) and 36.5% female (5874 with ASCVD, 4564 with DM) were included. The median follow-up time was 5.4 years. In the whole cohort, there were 11.9%, 5–10%, <1%, and <1% of the patients with missing data of CKD, lipid profiles, hypertension and HF, respectively. Those with prior ASCVD had more baseline CV risk factors than those without, including DM, CKD and HF. The patients with prior ASCVD also had more common use of statin and had lower LDL-C, lower non-HDL-C, lower HDL-C but similar TG and BMI levels, compared to those without ASCVD [Table 2].

**Table 2:** Baseline characteristics of the patients.

	Without ASCVD (T-PPARCLE)	With ASCVD (T-SPARCLE)	*p*
	N = 4859	N = 5874	
	Mean ± STD or %	Mean ± STD or %	
Age (years)	62.5 ± 11.8	65.6 ± 11.8	<0.01
Age ≥ 65	41.8%	52.6%	<0.01
Women	51.7%	24.0%	<0.01
TC (mg/dL)^a^	189.8 ± 38.6	170.0 ± 38.4	<0.01
HDL-C (mg/dL)	49.7 ± 15.3	44.6 ± 12.8	<0.01
LDL-C (mg/dL)^a^	111.4 ± 34.7	97.6 ± 34.1	<0.01
TG (mg/dL)^a^	146.1 ± 108.8	142.3 ± 97.2	0.06
Non-HDL-C (mg/dL)^a^	140.2 ± 37.9	124.7 ± 37.2	<0.01
Diabetes	37.9%	46.4%	<0.01
Hypertension^a^	82.9%	72.3%	<0.01
Chronic kidney disease^a^	19.6%	28.4%	<0.01
BMI (kg/m^2^)	26.4 ± 4.2	26.3 ± 4.2	0.16
Waist circumference (cm)	89.2 ± 10.8	92.1 ± 9.9	<0.01
Heart failure^a^	4.7%	12.2%	<0.01
Myocardial infarction	0.0%	68.7%	NA
Unstable angina	0.0%	3.2%	NA
Stroke	0.0%	18.7%	NA
PAOD	0.0%	2.4%	NA
PCI	0.0%	97.9%	NA
CABG	0.00%	2.6%	NA
Statin use	48.2%	69.1%	<0.01
Anti-platelet use	35.1%	82.9%	<0.01
MACE within 1 yr	0.37%	1.33%	<0.01

Abbreviations: BMI : body mass index; CABG : coronary artery bypass graft; MACE : major adverse cardiovascular events; PAOD : peripheral arterial occlusive disease; PCI : percutaneous coronary intervention; TC : Total cholesterol.

a Missing data: TG (n = 621, 5.8%), non-HDL-C (n = 1067, 9.9%), LDL-C (n = 843, 7.9%), hypertension (n = 18, 0.2%), chronic kidney disease (n = 1278, 11.9%), heart failure (n = 33, 0.3%), waist circumference (n = 1816, 16.9%).

**Fig. 1:**
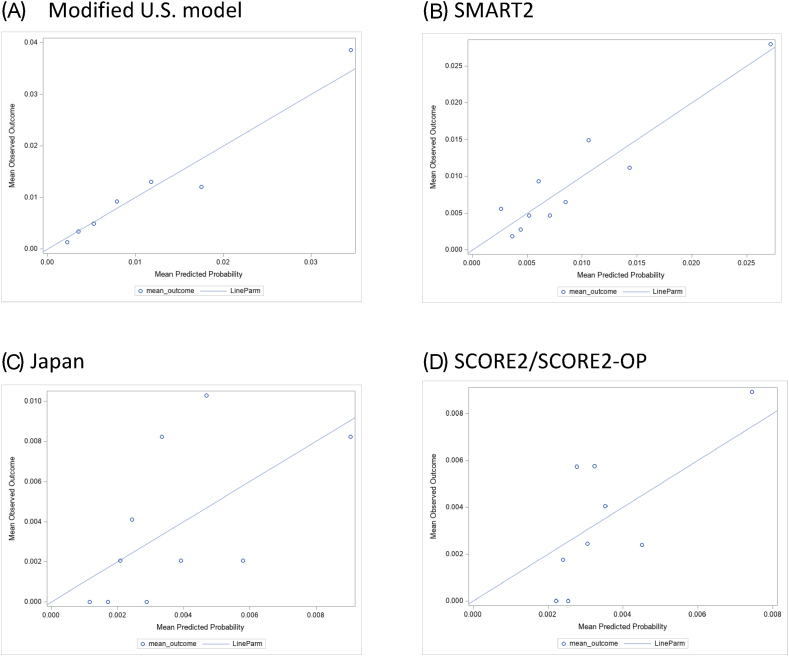
Calibration plots of different models.

Of the 10,733 patients, 96 of them had MACE in 1 year (1.0%). The MUS model analysis showed an AUC of 0.73 (0.68–0.78), Brier score of <0.01, Goodness-of-Fit test *p* = 0.67, while those of the SMART2 were 0.69 (0.64–0.75), <0.01 and 0.19, respectively. In those without ASCVD, Japan model analysis showed an AUC of 0.68 (0.58–0.78), Brier score of <0.01 and Goodness-of-Fit test *p* = 0.16 while those of the SCORE2 were 0.62 (0.52–0.77), <0.01 and 0.14, respectively [Table 3]. The calibration plots of each model are presented in [Fig. 1].

**Table 3:** The performance of various scoring systems to predict major adverse cardiovascular event outcomes in the entire study population or subgroups.

Risk score model	Population	N	AUC (95% CI)	Brier score (95% CI)	Goodness-of-Fit test (*p*-value)
Modified U.S. model	With or without ASCVD	10,733	0.73 (0.68–0.78)	<0.01	0.67
SMART2^a^	With or without ASCVD	10,733	0.69 (0.64–0.75)	<0.01	0.19
Japan^b^	Without ASCVD	4859	0.68 (0.58–0.78)	<0.01	0.16
SCORE2/SCORE2-OP	Without ASCVD, age 40–89	4366	0.62 (0.52–0.77)	<0.01	0.14

a In the SMART2 model, abdominal aneurysm, CRP level, years since ASCVD diagnosis were not included.

b In the Japan model, proteinuria and exercise were not included.

**Fig. 2:**
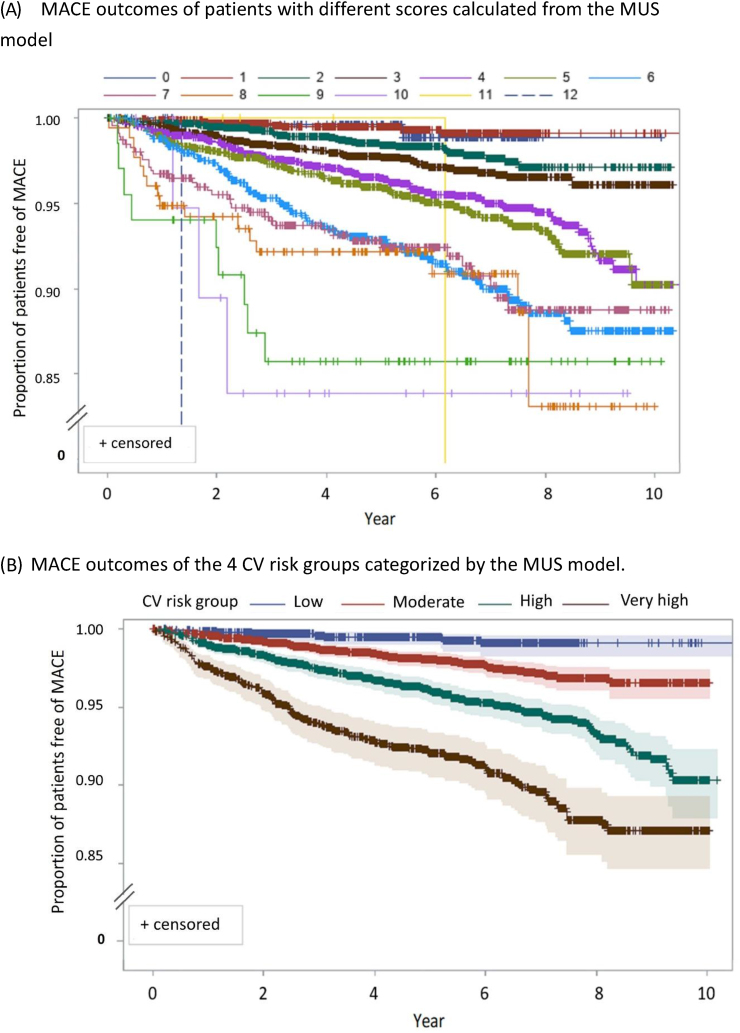
Modified US (MUS) model used to stratify different cardiovascular (CV) risk groups based on their major adverse cardiovascular event (MACE) outcomes.

**Fig. 3:**
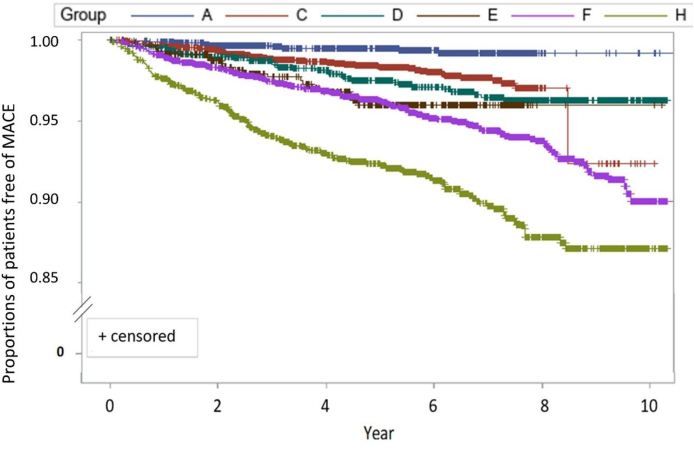
The major adverse cardiovascular event (MACE) outcomes of the 4 cardiovascular (CV) risk groups in [Fig. 1(B)] were further stratified based on the presence or absence of atherosclerotic cardiovascular disease (ASCVD). Group A: Low risk without ASCVD, C: Moderate risk without ASCVD, D: Moderate risk with ASCVD, E: High risk without ASCVD, F: High risk with ASCVD, H: Very high risk with ASCVD (Group B: Low risk with ASCVD and Group G: Very high risk without ASCVD; not presented due to low case number).

The MUS model could stratify the patients into four distinct risk groups [Fig. 2]: very high-risk (score ≥6), high-risk (score = 4–5), moderate-risk (score = 2–3) and low-risk (score = 0–1). The hazard ratio (HR) between different groups was calculated, with very high- vs. high-risk group HR = 1.91 (95% CI 1.53–2.39, *p* < 0.01), high- vs. moderate-risk group HR = 2.08 (95% CI 1.60–2.69, *p*< 0.01), and moderate- vs. low-risk group HR = 3.14 (95% CI 1.63–6.03, *p* < 0.01). 0.6% (n = 36) and 21.7% (n = 1272) of the patients with ASCVD were stratified into low and moderate risk groups, and 2.4% (n = 111) and 32.7% (n = 1494) of the patients with diabetes were stratified into low and moderate risk groups, respectively [Table 4]. In [Fig. 3], survival rates of individuals with and without a history of ASCVD are presented for each risk group. After adjusting for the MUS model, a history of ASCVD appeared not to be a significant predictor of adverse cardiovascular outcomes within each risk group.

**Table 4:** The characteristics and major adverse cardiovascular event outcomes of different cardiovascular risk groups.

CV risk group		Low (MUS-SCORE 0–1)	Moderate (MUS-SCORE 2–3)	High (MUS-SCORE 4–5)	Very high (MUS-SCORE ≧6)
N		1515	4083	3499	1636
With history of ASCVD		36 (2.4%)	1272 (31.2%)	2935 (83.9%)	1631 (99.7%)
With history of DM		111 (7.3%)	1494 (36.6)	1785 (51.0%)	1174 (71.8%)
TG ≥ 200 mg/dL		18.0%	17.8%	18.2%	17.7%
High non-HDL-C		9.0%	23.4%	41.8%	77.0%
High LDL-C		8.3%	23.8%	43.8%	82.5%
Low HDL-C		36.7%	41.1%	44.4%	48.0%
Outcome comparison with the lower level risk group (Cox regression)	HR		3.14	2.08	1.91
	95% CI		1.63–6.03	1.60–2.69	1.53–2.39
	*p*-value		<0.01	<0.01	<0.01

Abbreviations: ASCVD : atherosclerotic vascular disease; CV : cardiovascular; DM : diabetes mellitus, High non-HDL-C: non-HDL-C ≥100 mg/dL in very high-risk, ≥130 in high risk, ≥160 in moderate risk, ≥190 in low risk; High LDL-C: LDL-C ≥70 mg/dL in very high-risk group, ≥100 mg/dL in high-risk group, ≥130 mg/dL in moderate-risk group, ≥160 mg/dL in low-risk group; Low HDL-C, men ≤ 40 mg/dL, women ≤ 50 mg/dL.

## Discussion

The results of this study showed that the use of this simple MUS risk predicting model, based on the ACC/AHA treatment guideline, improved the precision of stratifying patients into different risk groups. These findings supported the Taiwanese guidelines, which were based on numerous studies conducted in Western populations [4,19], as well as the European guideline's concept of managing the patients based on the background risk factors rather than focusing solely on the primary or secondary prevention [5].

A simple model has several advantages. Firstly, according to the health belief model, the easy-to-use scoring system may enhance patients' risk perception, promote health behaviors, and facilitate shared decision making [12,20,21], as one cohort study showed that correct patient-perceived risk was associated with better cardiovascular preventive behaviors [22]. Secondly, the awareness-to-adherence model suggests that physicians may have better adherence to guidelines with a user-friendly model, leading to more accurate stratification of patients' risk [23]. Thirdly, it is more likely to generalize well in other populations or clinical settings [24]. Lastly, a simple model is more feasible to enroll patients for clinical trials.

In this study, overall, the various models tested demonstrated acceptable discrimination. The MUS model was able to accurately classify patients into different risk groups based on simple parameters. Some have proposed the Brier score as a better alternative to AUC because of better reliability and efficiency [25]. While there isn't a universally established threshold for a Brier score, it's generally accepted that a lower score signifies superior model performance. This premise is rooted in the fact that the Brier score measures the mean squared difference between predicted and actual outcomes; thus, a smaller score suggests a higher degree of accuracy in the model's predictions. However, interpreting the Brier score may be difficult due to the low event rate in our study. In situations of low prevalence, even a model with poor performance can achieve a low Brier score by simply predicting the majority class, in this case, the non-event [26]. The concept of miscalibration can be effectively assessed through the utilization of bothf the goodness-of-fit test and calibration plots. With the goodness-of-fit test, a *P*-value less than 0.05 generally indicates a substantial likelihood of miscalibration [27]. In our study, the calibration assessments were largely favorable. More specifically, the modified U.S. model demonstrated good calibration, suggesting its reliable performance in estimating cardiovascular risk within our study population.

The results of this study supported the risk assessment methods in both Western and Asian guidelines [3,4,28]. In the US guideline, patients with two major CV events were considered very high-risk [3]. In this study, those with two major CV events had a calculated score ≥4; these people could be further stratified into high- or very high-risk groups, and the target of lipid-lowering therapy may differ based on this stratification. Our results were consistent with some concepts of the European lipid treatment guideline, which suggested that management of patients should be based more on background risk factors rather than focusing solely on the primary or secondary prevention. However, our results also showed some important differences from those recommended in the European guideline [5]. In our study, a history of ASCVD did not necessarily indicate high risk of future MACE. All people with a history of ASCVD were categorized as very high risk in the European guideline, while about 24% of ASCVD patients were only categorized as moderate- or low-risk by the MUS model. In the European guideline, those with very high risk had an estimated CV mortality of 10% in 10 years [5]. In our cohort, the ASCVD patients with a 10-year major cardiovascular event rate were as follows: >10% in the very high-risk group, 5–10% in the high-risk group, 2–5% in the moderate-risk group, and ≤2% in the low-risk group. This difference, demonstrated in our study and previous Japanese REAL-CAD study [29], might reflect lower CV event rates in the Asian population. Diabetes is an important risk factor for future MACE. This risk, however, is not static but subject to change, especially considering recent advancements in the field of antidiabetic medications, which possess the potential to mitigate disease-related risks [30]. A notable observation was that approximately 40% of patients diagnosed with diabetes were categorized as low-to moderate-risk. This finding reinforces the recently emphasized recommendations from experts in diabetology advocating for a nuanced risk assessment in patients with diabetes [31,32]. This distinction is important, as it can guide personalized therapeutic decisions, paving the way towards a more targeted approach to diabetes management.

The results of this study are consistent with the US guideline, which suggests further risk stratification for both primary and secondary prevention to guide therapeutic strategies for further risk reduction in the statin era. The MUS model, which was adapted from the ACC/AHA 2018 guideline on lipid control, proved effective in stratifying the cardiovascular risk of patients, regardless of their ASCVD status, using only a few easily accessible parameters. Although our cohorts did not include patients with familial hypercholesterolemia, the modified model still performed well in this regard.

Multiple tools have been developed to assess CV risk worldwide. In Asians without ASCVD, the Framingham risk score was previously shown to be useful in predicting CV risk [7]. A study in a Korean population using the ACC/AHA 2013 pooled cohort equations (PCE) found an AUC score of 0.73, indicating good discrimination in patients free of ASCVD [9]. The MUS model developed in our study, however, performed well for patients with and without ASCVD. The SMART2 score, validated in Western populations with prior ASCVD, also performed well in our cohorts. However, the SMART2 score includes several continuous variables, making it less straightforward to stratify the CV risks of patients. Despite not including these continuous variables, the MUS model performed similarly to the SMART2 score in predicting CV risk for ASCVD patients and even better for those without ASCVD. Another risk model developed in Japan showed good discrimination with an AUC of 0.76 [18], but it included the variable of proteinuria, which was not recorded in our cohorts. Incorporating such variables could further improve the performance of the MUS model.

This study had some limitations that should be considered. Firstly, the patients included in the registry cohorts had underlying risk factors and were receiving medical treatment, which might have resulted in the exclusion of individuals with extremely low CV risk, potentially affecting the performance of the MUS model. Secondly, given that the data pool for model learning and prediction was consequently diminished due to low event rates and shorter follow-up periods, this might have led to lower AUC values [33,34]. Future studies with longer follow-up and inclusion of healthier individuals are needed. Thirdly, our study design considered the risk of Type I error, often linked with multiple comparisons in survival analyses. Instead, we evaluated each model based on its unique features and target demographics. This approach could circumvent the potential issues of direct model comparisons, facilitating a more nuanced and context-specific interpretation of each model's application [35]. Fourthly, our study did not address the issue of arrhythmia in patients with ASCVD or heart failure. Also, we did not record the use of device therapy, such as cardiac resynchronization therapy which have been suggested to influence clinical outcomes in diabetes patients [31,36]. Finally, incorporating genetic information, biomarkers, and imaging may improve the power of risk prediction [[37], [38], [39], [40]]. Similarly, the effect of novel therapeutic agents like sacubitril/valsartan and sodium/glucose cotransporter-2 inhibitors (SGLT2i), which have shown promise in altering the course of conditions such as heart failure and diabetes mellitus, was not thoroughly documented in our study [15]. However, the MUS model's simplicity, using only a few easily accessible parameters, made it user-friendly for both patients and physicians. Nevertheless, future studies should validate this model in other populations and explore the potential benefits of incorporating additional clinical and genetic information to enhance risk prediction.

In conclusion, the MUS model, which was based on the US ACC/AHA 2018 guideline, proved to be a practical risk stratification tool for both Asian patients with and without ASCVD. The model demonstrated accurate predictions of major cardiovascular events and was found to be comparable or superior to other established risk models. Its simplicity and reliance on easily available parameters make it a useful option for both Asian patients and physicians. Additionally, the study implies that patient management should be based on their background risk factors rather than solely focusing on primary or secondary prevention.

## Funding

This study is supported by Taiwan Society of Lipids & Atherosclerosis since 2009, Taiwan Ministry of Science and Technology since 2012 (Project code: NRPB-TR11: 100-2325-B-002-075, 102-2325-B-002-097, 104-2325-B-002-036, 105-2325-B-002-035, 106-2321-B-002-029, 107-2321-B-002-035, 108-2321-B-002-043, 109-2321-B-002-039, and 110-2745-B-002 -004), and Taiwan Association of Lipid Educators since 2018.

## Declaration of generative AI and AI-assisted technologies in the writing process

During the preparation of this work, the authors used ChatGPT in order to improve language and readability. After using this tool/service, the authors reviewed and edited the content as needed and took full responsibility for the content of the publication.

## Conflicts of interest

The authors declared no conflicts of interest.

## References

[1] Vos T., Lim S.S., Abbafati C., Abbas K.M., Abbasi M., Abbasifard M. (2020). Global burden of 369 diseases and injuries in 204 countries and territories, 1990-2019: a systematic analysis for the Global Burden of Disease Study 2019. Lancet.

[2] Ueshima H., Sekikawa A., Miura K., Turin T.C., Takashima N., Kita Y. (2008). Cardiovascular disease and risk factors in Asia: a selected review. Circulation.

[3] Grundy S.M., Stone N.J., Bailey A.L., Beam C., Birtcher K.K., Blumenthal R.S. (2019). AHA/ACC/AACVPR/AAPA/ABC/ACPM/ADA/AGS/APhA/ASPC/NLA/PCNA guideline on the management of blood cholesterol:A Report of the American College of Cardiology/American Heart Association Task Force on Clinical Practice Guidelines. J Am Coll Cardiol.

[4] Li Y.H., Ueng K.C., Jeng J.S., Charng M.J., Lin T.H., Chien K.-L. (2017). 2017 Taiwan lipid guidelines for high risk patients. J Formos Med Assoc.

[5] Mach F., Baigent C., Catapano A.L., Koskinas K.C., Casula M., Badimon L. (2019). 2019 ESC/EAS Guidelines for the management of dyslipidaemias: lipid modification to reduce cardiovascular risk: the Task Force for the management of dyslipidaemias of the European Society of Cardiology (ESC) and European Atherosclerosis Society (EAS). Eur Heart J.

[6] Barzi F., Patel A., Gu D., Sritara P., Lam T.H., Rodgers A. (2007). Cardiovascular risk prediction tools for populations in Asia. J Epidemiol Community Health.

[7] Selvarajah S., Kaur G., Haniff J., Cheong K.C., Hiong T.G., van der Graaf Y. (2014). Comparison of the Framingham Risk Score, SCORE and WHO/ISH cardiovascular risk prediction models in an Asian population. Int J Cardiol.

[8] Liu J., Hong Y., D’Agostino S., Ralph B., Wu Z., Wang W. (2004). Predictive value for the Chinese population of the Framingham CHD risk assessment tool compared with the Chinese multi-provincial cohort study. JAMA.

[9] Jung K.J., Jang Y., Oh D.J., Oh B.-H., Lee S.H., Park S.W. (2015). The ACC/AHA 2013 pooled cohort equations compared to a Korean Risk Prediction Model for atherosclerotic cardiovascular disease. Atherosclerosis.

[10] Magnani J.W., Mujahid M.S., Aronow H.D., Cené C.W., Dickson V.V., Havranek E. (2018). Health literacy and cardiovascular disease: fundamental relevance to primary and secondary prevention: a scientific statement from the American Heart Association. Circulation.

[11] Glanz K(Ed), Rimer BK(Ed) (2008).

[12] Janz N.K., Becker M.H. (1984). The health belief model: a decade later. Health Educ Q.

[13] Dorresteijn J.A.N., Visseren F.L.J., Wassink A.M.J., Gondrie M.J.A., Steyerberg E.W., Ridker P.M. (2013). Development and validation of a prediction rule for recurrent vascular events based on a cohort study of patients with arterial disease: the SMART risk score. Heart.

[14] Hageman S.H.J., McKay A.J., Ueda P., Gunn L.H., Jernberg T., Hagström E. (2022). Estimation of recurrent atherosclerotic cardiovascular event risk in patients with established cardiovascular disease: the updated SMART2 algorithm. Eur Heart J.

[15] Visseren F.L.J., Mach F., Smulders Y.M., Carballo D., Koskinas K.C., Bäck M. (2021). 2021 ESC Guidelines on cardiovascular disease prevention in clinical practice: developed by the Task Force for cardiovascular disease prevention in clinical practice with representatives of the European Society of Cardiology and 12 medical societies with the special contribution of the European Association of Preventive Cardiology (EAPC). Eur Heart J.

[16] Collet J.P., Thiele H., Barbato E., Barthélémy O., Bauersachs J., Bhatt D.L. (2021). 2020 ESC Guidelines for the management of acute coronary syndromes in patients presenting without persistent ST-segment elevation. Eur Heart J.

[17] Global Health Estimates: Life expectancy and leading causes of death and disability; 2019,https://www.who.int/data/gho/data/themes/mortality-and-global-health-estimates/;2022 [accessed 20 March 2022].

[18] Honda T., Chen S., Hata J., Yoshida D., Hirakawa Y., Furuta Y. (2022). Development and validation of a risk prediction model for atherosclerotic cardiovascular disease in Japanese adults: the Hisayama study. J Atheroscler Thromb.

[19] Ueng K.C., Chiang C.E., Chao T.H., Wu Y.W., Lee W.L., Li Y.H. (2023). 2023 guidelines of the Taiwan Society of Cardiology on the diagnosis and management of chronic coronary syndrome. Acta Cardiol Sin.

[20] Ferrer R., Klein W.M. (2015). Risk perceptions and health behavior. Curr Opin Psychol.

[21] Elwyn G., Frosch D., Thomson R., Joseph-Williams N., Lloyd A., Kinnersley P. (2012). Shared decision making: a model for clinical practice. J Gen Intern Med.

[22] Navar A.M., Wang T.Y., Li S., Mi X., Li Z., Robinson J.G. (2021). Patient-perceived versus actual risk of cardiovascular disease and associated willingness to consider and use prevention therapy. Circ Cardiovasc Qual Outcomes.

[23] Pathman D.E., Konrad T.R., Freed G.L., Freeman V.A., Koch G.G. (1996). The awareness-to-adherence model of the steps to clinical guideline compliance. The case of pediatric vaccine recommendations. Med Care.

[24] Lever J., Krzywinski M., Altman N. (2016). Model selection and overfitting. Nat Methods.

[25] Wang X., Claggett B.L., Tian L., Malachias M.V.B., Pfeffer M.A., Wei L.-J. (2023). Quantifying and interpreting the prediction accuracy of models for the time of a cardiovascular event—moving beyond C statistic: a review. JAMA Cardiol.

[26] Assel M., Sjoberg D.D., Vickers A.J. (2017). The Brier score does not evaluate the clinical utility of diagnostic tests or prediction models. Diagn Progn Res.

[27] Demler O.V., Paynter N.P., Cook N.R. (2015). Tests of calibration and goodness-of-fit in the survival setting. Stat Med.

[28] Kinoshita M., Yokote K., Arai H., Iida M., Ishigaki Y., Ishibashi S. (2018). Japan Atherosclerosis Society (JAS) guidelines for prevention of atherosclerotic cardiovascular diseases 2017. J Atheroscler Thromb.

[29] Taguchi I., Iimuro S., Iwata H., Takashima H., Abe M., Amiya E. (2018). High-dose versus low-dose pitavastatin in Japanese patients with stable coronary artery disease (REAL-CAD). Circulation.

[30] Joseph J.J., Deedwania P., Acharya T., Aguilar D., Bhatt D.L., Chyun D.A. (2022). Comprehensive management of cardiovascular risk factors for adults with type 2 diabetes: a scientific statement from the American Heart Association. Circulation.

[31] Cosentino F., Grant P.J., Aboyans V., Bailey C.J., Ceriello A., Delgado V. (2020). 2019 ESC Guidelines on diabetes, pre-diabetes, and cardiovascular diseases developed in collaboration with the EASD: the Task Force for diabetes, pre-diabetes, and cardiovascular diseases of the European Society of Cardiology (ESC) and the European Association for the Study of Diabetes (EASD). Eur Heart J.

[32] Bertoluci M.C., Rocha V.Z. (2017). Cardiovascular risk assessment in patients with diabetes. Diabetol Metab Syndrome.

[33] Seiffert C, Khoshgoftaar TM, Hulse JV, Napolitano A. Mining data with rare events: a case study. 19th IEEE International Conference on Tools with Artificial Intelligence(ICTAI 2007) 2007;2:p.132–139.

[34] Hulse J.V., Khoshgoftaar T.M., Napolitano A. (2007). Proceedings of the 24th international conference on Machine learning.

[35] Lee S., Lee D.K. (2018). What is the proper way to apply the multiple comparison test?. Korean J Anesthesiol.

[36] Sardu C., Barbieri M., Santamaria M., Giordano V., Sacra C., Paolisso P. (2017). Multipolar pacing by cardiac resynchronization therapy with a defibrillators treatment in type 2 diabetes mellitus failing heart patients: impact on responders rate, and clinical outcomes. Cardiovasc Diabetol.

[37] O’Sullivan J.W., Raghavan S., Marquez-Luna C., Luzum J.A., Damrauer S.M., Ashley E.A. (2022). Polygenic risk scores for cardiovascular disease: a scientific statement from the American Heart Association. Circulation.

[38] Gui L., Wu F., Han X., Dai X., Qiu G., Li J. (2014). A multilocus genetic risk score predicts coronary heart disease risk in a Chinese Han population. Atherosclerosis.

[39] Saraste A., Barbato E., Capodanno D., Edvardsen T., Prescott E., Achenbach S. (2019). Imaging in ESC clinical guidelines: chronic coronary syndromes. Eur Heart J Cardiovasc Imaging.

[40] Tamarappoo B.K., Lin A., Commandeur F., McElhinney P.A., Cadet S., Goeller M. (2021). Machine learning integration of circulating and imaging biomarkers for explainable patient-specific prediction of cardiac events: a prospective study. Atherosclerosis.

